# Stable polymorph of morphine^1^

**DOI:** 10.1107/S1600536812048945

**Published:** 2012-12-05

**Authors:** Thomas Gelbrich, Doris E. Braun, Ulrich J. Griesser

**Affiliations:** a Institute of Pharmacy, University of Innsbruck, Innrain 52c, 6020 Innsbruck, Austria

## Abstract

In the stable polymorph of the title compound, C_17_H_19_NO_3_ [systematic name: (5α,6α)-7,8-didehydro-4,5-ep­oxy-17-methyl­morphinan-3,6-diol], the mol­ecular conformation is in agreement with the characteristics of previously reported morphine forms. The molecule displays the typical T-shape and its piperidine ring adopts a slightly distorted chair conformation. Inter­molecular O—H⋯O hydrogen bonds link the mol­ecules into helical chains parallel to the *b* axis. Intra­molecular O—H⋯O hydrogen bonds are also observed.

## Related literature

For related structures, see: Guguta *et al.* (2008 ▶); Gylbert (1973 ▶); Mackay & Hodgkin (1955 ▶); Bye (1976 ▶); Wongweichintana *et al.* (1984 ▶); Lutz & Spek (1998 ▶); Scheins *et al.* (2005 ▶); Gelbrich *et al.* (2012 ▶). For decriptions of morphine polymorphs, see: Kofler (1933 ▶); Kuhnert-Brandstätter *et al.* (1975 ▶). For a description of the Cambridge Structural Database, see: Allen (2002 ▶). For the program *XPac*, see: Gelbrich & Hursthouse (2005 ▶).
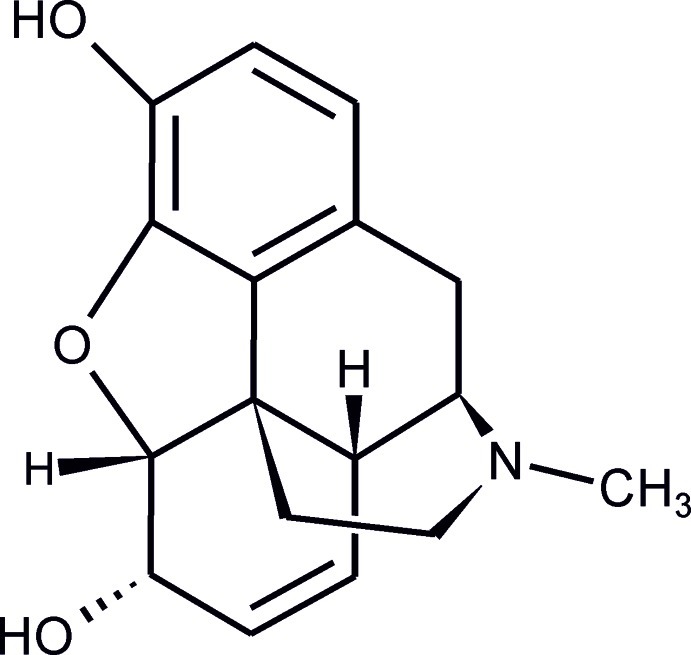


## Experimental

### 

#### Crystal data

C_17_H_19_NO_3_
*M* *_r_* = 285.33
Orthorhombic, 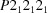

*a* = 7.6989 (10) Å
*b* = 12.737 (4) Å
*c* = 13.740 (4) Å
*V* = 1347.4 (6) Å^3^
*Z* = 4
Cu *K*α radiation
μ = 0.78 mm^−1^
*T* = 173 K
0.15 × 0.10 × 0.03 mm

#### Data collection

Oxford Diffraction Xcalibur (Ruby, Gemini ultra) diffractometer
Absorption correction: multi-scan (*CrysAlis PRO*; Oxford Diffraction, 2003 ▶) *T*_min_ = 0.624, *T*_max_ = 1.000
13009 measured reflections
1408 independent reflections
977 reflections with *I* > 2σ(*I*)
*R*_int_ = 0.118

#### Refinement

*R*[*F* ^2^ > 2σ(*F* ^2^)] = 0.068
*wR*(*F* ^2^) = 0.141
*S* = 1.01
1408 reflections
192 parameters
H-atom parameters constrained
Δρ_max_ = 0.27 e Å^−3^
Δρ_min_ = −0.26 e Å^−3^

### 

Data collection: *CrysAlis PRO* (Oxford Diffraction, 2003 ▶); cell refinement: *CrysAlis PRO*; data reduction: *CrysAlis PRO*; program(s) used to solve structure: *SHELXS97* (Sheldrick, 2008 ▶); program(s) used to refine structure: *SHELXL97* (Sheldrick, 2008 ▶); molecular graphics: *XP* in *SHELXTL* (Bruker, 1998 ▶) and *Mercury* (Bruno *et al.*, 2002 ▶); software used to prepare material for publication: *publCIF* (Westrip, 2010 ▶).

## Supplementary Material

Crystal structure: contains datablock(s) I, global. DOI: 10.1107/S1600536812048945/im2413sup1.cif

Structure factors: contains datablock(s) I. DOI: 10.1107/S1600536812048945/im2413Isup2.hkl

Additional supplementary materials:  crystallographic information; 3D view; checkCIF report

## Figures and Tables

**Table 1:** Hydrogen-bond geometry (Å, °)

*D*—H⋯*A*	*D*—H	H⋯*A*	*D*⋯*A*	*D*—H⋯*A*
O1—H1⋯O3^i^	0.84	1.96	2.757 (6)	159
O3—H3⋯O2	0.84	2.17	2.629 (6)	114

Symmetry code: (i) 
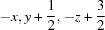
.

**Fig. 1.**
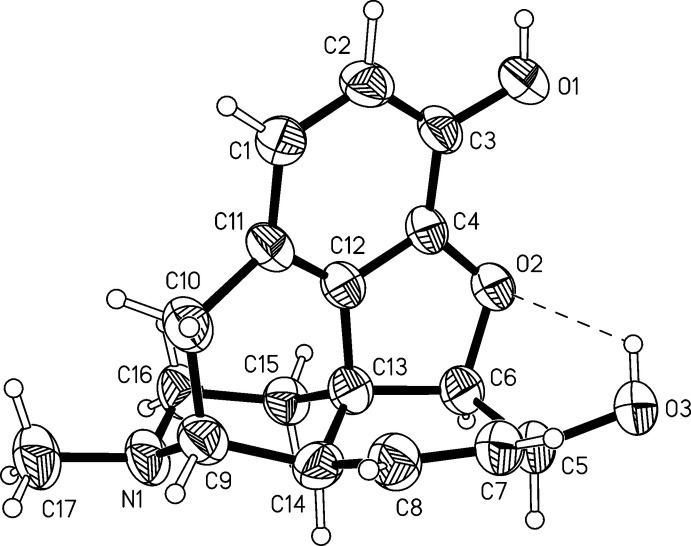
Molcular structure of morphine with displacement ellipsoids are drawn at the 50% probability level and hydrogen atoms shown as spheres of arbitrary size.

**Fig. 2.**
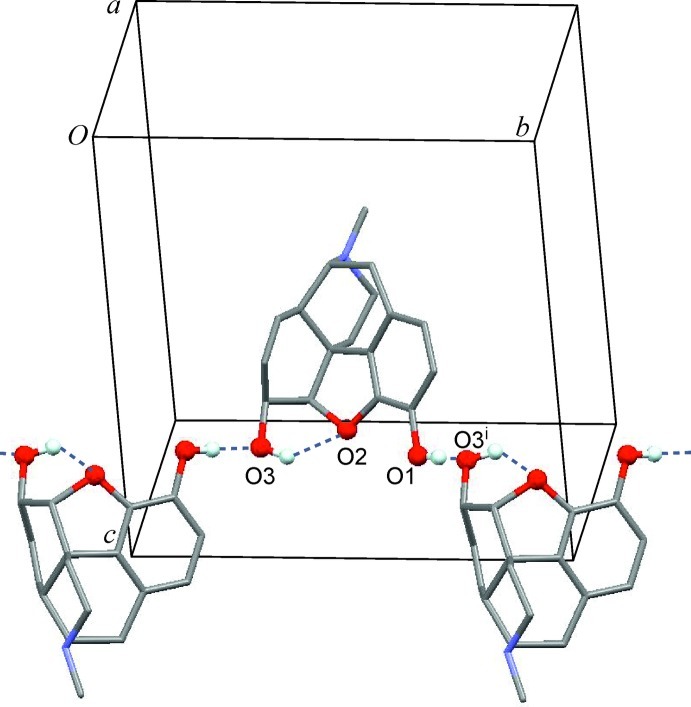
Hydrogen bonded helical chain. Intramolecular and intermolecular O—H···O bonds are drawn as broken lines; H and O atoms involved in hydrogen bonding are drawn as balls [symmetry code: (i) -x, y + 1/2, -z + 3/2.].

## supplementary crystallographic information

### Comment

Morphine is the main alkaloid of opium, the dried latex of the opium poppy (*Papaver somniferum*). The Cambridge Structural Database (CSD; version 5.33 and updates; Allen, 2002) contains a number of free base and salt structures of morphine: a monohydrate (Bye, 1976), a hydrochloride trihydrate (Gylbert, 1973), a hydroiodide dihydrate (Mackay & Hodgkin, 1955), a complex with β-phenylhydracrylic acid (Lutz & Spek, 1998) and a bis(morphinium) dihydrogensulfate pentahydrate (Wongweichintana *et al.*, 1984). A hydrochloride anhydrate structure was recently reported by us (Gelbrich *et al.*, 2012). The title structure was previously solved from powder data by Guguta *et al.* (2008), however the corresponding atomic coordinates are not available from the CSD or from supplementary materials accompanying this report.

According to Kofler (1933), morphine can exist in two distinct polymorphic modifications, and the characteristics of the crystals investigated by us match Kofler's description of the stable form. Our thermomicroscopy experiments have shown that the investigated crystals melt under decomposition at 254 °C (the applied heating rate was 5 °C per minute). This behaviour is in agreement with reports given by Kofler (1933) and Kuhnert-Brandstätter *et al.* (1975).

The geometry of the molecular morphine scaffold (Figure 1) with its five rings agrees with the characteristics of the related salt and free base structures mentioned above. The title structure displays two sets of O—H···O bonds, one of which is intramolecular and the other is intermolecular (Table 1). Intermolecular hydrogen bonds link the morphine molecules into an infinite helical chain that propagates parallel to the *b*-axis (Figure 2).

The packing of the geometrically inflexible morphine moieties in the title structure was compared with corresponding packing arrangements present in the six morphine forms mentioned above (Bye, 1976; Gelbrich *et al.*, 2012; Gylbert, 1973; Mackay & Hodgkin, 1955; Lutz & Spek, 1998; Wongweichintana *et al.*, 1984), using the program *XPac* (Gelbrich & Hursthouse, 2005). These comparisons have shown that the packing mode of morphine molecules in the stable form is unique and has no supramolecular constructs in common with any of the other structures in this group.

### Experimental

Morphine was obtained from Heilmittelwerke Wien, Austria. Very thin, plate-shaped crystals of the stable polymorph were yielded from a sublimation experiment carried out on a Kofler hot bench at approximately 150 °C, using two glass slides separated by a spacer ring of 1 cm height.

### Refinement

All H atoms were identified in a difference map. Methyl H atoms were idealized and included as rigid groups allowed to rotate but not tip (C—H = 0.98 Å) and H atoms bonded to oxygen atoms (O—H = 0.84 Å), tertiary CH (C—H = 0.99 Å), secondary CH_2_ (C—H = 0.99 Å) and aromatic carbon atoms (C—H = 0.95 Å) were positioned geometrically. The temperature parameters of the methyl H atoms were set to *U*_iso_(H) = 1.5 *U*_eq_(C) of the parent carbon atom, for all other H atoms they were set to *U*_iso_(H) = 1.2 *U*_eq_(C or O).

### Crystal data

**Table:**

C_17_H_19_NO_3_	*F*(000) = 608
*M**_r_* = 285.33	*D*_x_ = 1.407 Mg m^−^^3^
Orthorhombic, *P*2_1_2_1_2_1_	Cu *K*α radiation, λ = 1.54180 Å
Hall symbol: P 2ac 2ab	Cell parameters from 1360 reflections
*a* = 7.6989 (10) Å	θ = 3.2–68.2°
*b* = 12.737 (4) Å	µ = 0.78 mm^−^^1^
*c* = 13.740 (4) Å	*T* = 173 K
*V* = 1347.4 (6) Å^3^	Plate, colourless
*Z* = 4	0.15 × 0.10 × 0.03 mm

### Data collection

**Table:**

Oxford Diffraction Xcalibur (Ruby, Gemini ultra) diffractometer	1408 independent reflections
Radiation source: Enhance (Mo) X-ray Source	977 reflections with *I* > 2σ(*I*)
Graphite monochromator	*R*_int_ = 0.118
Detector resolution: 10.3575 pixels mm^-1^	θ_max_ = 68.2°, θ_min_ = 4.7°
ω scans	*h* = −9→8
Absorption correction: multi-scan (*CrysAlis PRO*; Oxford Diffraction, 2003)	*k* = −14→15
*T*_min_ = 0.624, *T*_max_ = 1.000	*l* = −16→16
13009 measured reflections	

### Refinement

**Table:**

Refinement on *F*^2^	Secondary atom site location: difference Fourier map
Least-squares matrix: full	Hydrogen site location: inferred from neighbouring sites
*R*[*F*^2^ > 2σ(*F*^2^)] = 0.068	H-atom parameters constrained
*wR*(*F*^2^) = 0.141	*w* = 1/[σ^2^(*F*_o_^2^) + (0.0112*P*)^2^ + 2.*P*] where *P* = (*F*_o_^2^ + 2*F*_c_^2^)/3
*S* = 1.01	(Δ/σ)_max_ < 0.001
1408 reflections	Δρ_max_ = 0.27 e Å^−^^3^
192 parameters	Δρ_min_ = −0.26 e Å^−^^3^
0 restraints	Extinction correction: *SHELXL97* (Sheldrick, 2008), Fc^*^=kFc[1+0.001xFc^2^λ^3^/sin(2θ)]^-1/4^
Primary atom site location: structure-invariant direct methods	Extinction coefficient: 0.0043 (4)

### Special details

**Table:**

Geometry. All e.s.d.'s (except the e.s.d. in the dihedral angle between two l.s. planes) are estimated using the full covariance matrix. The cell e.s.d.'s are taken into account individually in the estimation of e.s.d.'s in distances, angles and torsion angles; correlations between e.s.d.'s in cell parameters are only used when they are defined by crystal symmetry. An approximate (isotropic) treatment of cell e.s.d.'s is used for estimating e.s.d.'s involving l.s. planes.
Refinement. Refinement of *F*^2^ against ALL reflections. The weighted *R*-factor *wR* and goodness of fit *S* are based on *F*^2^, conventional *R*-factors *R* are based on *F*, with *F* set to zero for negative *F*^2^. The threshold expression of *F*^2^ > σ(*F*^2^) is used only for calculating *R*-factors(gt) *etc*. and is not relevant to the choice of reflections for refinement. *R*-factors based on *F*^2^ are statistically about twice as large as those based on *F*, and *R*- factors based on ALL data will be even larger.The Flack *x* parameter (Flack, 1983) and the Hooft *y* parameter (Hooft *et al.*, 2008) were both indeterminate due to a lack of significant resonant scattering. Accordingly, Friedel opposites were merged prior to the final refinement. [Flack, H. D. (1983). *Acta Cryst.* A39, 876–881; Hooft, R. W. W., Straver, L. H. & Spek, A. L. (2008). *J. Appl. Cryst.*41, 96–103.]

### Fractional atomic coordinates and isotropic or equivalent isotropic displacement parameters (Å^2^)

**Table:**

	*x*	*y*	*z*	*U*_iso_*/*U*_eq_	
O1	0.0998 (5)	0.6564 (3)	0.7856 (3)	0.0520 (12)	
H1	0.0431	0.7103	0.7705	0.062*	
O2	0.3165 (5)	0.4742 (3)	0.7958 (3)	0.0466 (11)	
O3	0.1429 (6)	0.2969 (3)	0.7838 (3)	0.0546 (12)	
H3	0.1096	0.3587	0.7941	0.066*	
N1	0.8422 (6)	0.4378 (4)	0.5555 (4)	0.0492 (14)	
C1	0.3549 (8)	0.6662 (5)	0.5563 (5)	0.0458 (15)	
H1A	0.3611	0.7068	0.4984	0.055*	
C2	0.2382 (8)	0.6935 (5)	0.6290 (5)	0.0466 (16)	
H2	0.1665	0.7534	0.6198	0.056*	
C3	0.2223 (7)	0.6362 (5)	0.7147 (5)	0.0429 (15)	
C4	0.3281 (7)	0.5499 (4)	0.7238 (5)	0.0408 (14)	
C5	0.2959 (8)	0.2985 (5)	0.7233 (5)	0.0494 (17)	
H5	0.3554	0.2293	0.7320	0.059*	
C6	0.4208 (7)	0.3836 (5)	0.7615 (5)	0.0462 (16)	
H6	0.4902	0.3548	0.8168	0.055*	
C7	0.2554 (9)	0.3091 (5)	0.6180 (5)	0.0499 (17)	
H7	0.1398	0.2980	0.5961	0.060*	
C8	0.3781 (9)	0.3338 (5)	0.5537 (5)	0.0506 (17)	
H8	0.3524	0.3340	0.4861	0.061*	
C9	0.6652 (8)	0.4267 (5)	0.5160 (5)	0.0450 (15)	
H9	0.6748	0.3841	0.4551	0.054*	
C10	0.5732 (8)	0.5321 (5)	0.4878 (5)	0.0491 (17)	
H10A	0.6630	0.5838	0.4686	0.059*	
H10B	0.4984	0.5194	0.4304	0.059*	
C11	0.4634 (7)	0.5792 (5)	0.5682 (4)	0.0410 (14)	
C12	0.4530 (7)	0.5257 (5)	0.6546 (4)	0.0386 (14)	
C13	0.5465 (8)	0.4258 (5)	0.6810 (4)	0.0415 (15)	
C14	0.5584 (8)	0.3617 (5)	0.5888 (5)	0.0466 (16)	
H14	0.6227	0.2953	0.6033	0.056*	
C15	0.7290 (7)	0.4477 (5)	0.7212 (5)	0.0443 (15)	
H15A	0.7199	0.4948	0.7783	0.053*	
H15B	0.7818	0.3809	0.7432	0.053*	
C16	0.8453 (8)	0.4978 (5)	0.6457 (5)	0.0521 (17)	
H16A	0.8056	0.5704	0.6329	0.062*	
H16B	0.9657	0.5012	0.6708	0.062*	
C17	0.9652 (8)	0.4788 (6)	0.4841 (5)	0.060 (2)	
H17A	0.9335	0.5510	0.4672	0.090*	
H17B	0.9621	0.4350	0.4254	0.090*	
H17C	1.0826	0.4778	0.5117	0.090*	

### Atomic displacement parameters (Å^2^)

**Table:**

	*U*^11^	*U*^22^	*U*^33^	*U*^12^	*U*^13^	*U*^23^
O1	0.040 (2)	0.053 (2)	0.063 (3)	0.012 (2)	0.008 (2)	0.002 (2)
O2	0.031 (2)	0.048 (2)	0.060 (3)	0.0019 (19)	0.004 (2)	0.000 (2)
O3	0.040 (2)	0.050 (2)	0.074 (3)	−0.006 (2)	0.014 (3)	−0.002 (2)
N1	0.026 (2)	0.063 (3)	0.059 (3)	0.001 (3)	0.005 (2)	0.003 (3)
C1	0.040 (3)	0.043 (3)	0.055 (4)	0.003 (3)	0.002 (3)	0.006 (3)
C2	0.035 (3)	0.044 (3)	0.061 (5)	0.008 (3)	−0.004 (3)	0.000 (3)
C3	0.027 (3)	0.047 (3)	0.055 (4)	0.003 (3)	0.003 (3)	−0.003 (3)
C4	0.025 (3)	0.046 (3)	0.051 (4)	0.000 (3)	0.001 (3)	0.000 (3)
C5	0.034 (3)	0.048 (4)	0.066 (5)	−0.006 (3)	0.009 (3)	−0.001 (3)
C6	0.030 (3)	0.048 (4)	0.061 (4)	0.000 (3)	−0.001 (3)	0.003 (3)
C7	0.039 (3)	0.047 (4)	0.064 (5)	−0.005 (3)	0.006 (3)	−0.005 (3)
C8	0.044 (3)	0.052 (4)	0.056 (4)	−0.005 (3)	0.003 (3)	−0.008 (3)
C9	0.037 (3)	0.048 (4)	0.050 (4)	0.004 (3)	0.003 (3)	−0.003 (3)
C10	0.040 (3)	0.058 (4)	0.050 (4)	0.008 (3)	0.007 (3)	0.006 (3)
C11	0.030 (3)	0.047 (3)	0.045 (4)	0.004 (3)	0.003 (3)	−0.006 (3)
C12	0.024 (3)	0.044 (3)	0.048 (4)	0.002 (3)	−0.003 (3)	0.001 (3)
C13	0.030 (3)	0.046 (3)	0.048 (4)	0.000 (3)	−0.002 (3)	0.000 (3)
C14	0.038 (3)	0.038 (3)	0.065 (5)	−0.001 (3)	−0.001 (3)	0.002 (3)
C15	0.032 (3)	0.051 (4)	0.050 (4)	0.001 (3)	−0.005 (3)	0.000 (3)
C16	0.028 (3)	0.064 (4)	0.064 (4)	−0.003 (3)	0.000 (3)	0.000 (4)
C17	0.033 (3)	0.080 (5)	0.066 (5)	0.000 (4)	0.008 (3)	0.008 (4)

### Geometric parameters (Å, º)

**Table:**

O1—C3	1.380 (7)	C7—H7	0.9500
O1—H1	0.8400	C8—C14	1.512 (9)
O2—C4	1.385 (7)	C8—H8	0.9500
O2—C6	1.483 (7)	C9—C14	1.538 (9)
O3—C5	1.442 (8)	C9—C10	1.566 (9)
O3—H3	0.8400	C9—H9	1.0000
N1—C16	1.456 (8)	C10—C11	1.516 (8)
N1—C17	1.460 (8)	C10—H10A	0.9900
N1—C9	1.474 (8)	C10—H10B	0.9900
C1—C2	1.387 (9)	C11—C12	1.371 (8)
C1—C11	1.397 (8)	C12—C13	1.507 (8)
C1—H1A	0.9500	C13—C14	1.511 (9)
C2—C3	1.390 (9)	C13—C15	1.535 (8)
C2—H2	0.9500	C14—H14	1.0000
C3—C4	1.374 (8)	C15—C16	1.512 (8)
C4—C12	1.387 (8)	C15—H15A	0.9900
C5—C7	1.486 (9)	C15—H15B	0.9900
C5—C6	1.542 (8)	C16—H16A	0.9900
C5—H5	1.0000	C16—H16B	0.9900
C6—C13	1.563 (9)	C17—H17A	0.9800
C6—H6	1.0000	C17—H17B	0.9800
C7—C8	1.331 (9)	C17—H17C	0.9800
			
C3—O1—H1	109.5	C11—C10—C9	114.3 (5)
C4—O2—C6	106.2 (4)	C11—C10—H10A	108.7
C5—O3—H3	109.5	C9—C10—H10A	108.7
C16—N1—C17	112.0 (5)	C11—C10—H10B	108.7
C16—N1—C9	112.2 (5)	C9—C10—H10B	108.7
C17—N1—C9	112.7 (5)	H10A—C10—H10B	107.6
C2—C1—C11	120.1 (6)	C12—C11—C1	117.4 (6)
C2—C1—H1A	119.9	C12—C11—C10	117.9 (5)
C11—C1—H1A	119.9	C1—C11—C10	124.2 (6)
C1—C2—C3	122.3 (6)	C11—C12—C4	121.6 (5)
C1—C2—H2	118.8	C11—C12—C13	126.9 (5)
C3—C2—H2	118.8	C4—C12—C13	110.7 (5)
C4—C3—O1	119.3 (6)	C12—C13—C14	106.5 (5)
C4—C3—C2	116.4 (6)	C12—C13—C15	111.7 (5)
O1—C3—C2	124.1 (5)	C14—C13—C15	110.1 (5)
C3—C4—O2	125.7 (5)	C12—C13—C6	99.5 (5)
C3—C4—C12	121.7 (6)	C14—C13—C6	116.4 (5)
O2—C4—C12	112.4 (5)	C15—C13—C6	112.0 (5)
O3—C5—C7	113.0 (5)	C13—C14—C8	109.8 (5)
O3—C5—C6	108.9 (5)	C13—C14—C9	106.7 (5)
C7—C5—C6	113.5 (5)	C8—C14—C9	114.2 (6)
O3—C5—H5	107.0	C13—C14—H14	108.7
C7—C5—H5	107.0	C8—C14—H14	108.7
C6—C5—H5	107.0	C9—C14—H14	108.7
O2—C6—C5	108.5 (5)	C16—C15—C13	111.9 (5)
O2—C6—C13	107.1 (5)	C16—C15—H15A	109.2
C5—C6—C13	112.8 (5)	C13—C15—H15A	109.2
O2—C6—H6	109.5	C16—C15—H15B	109.2
C5—C6—H6	109.5	C13—C15—H15B	109.2
C13—C6—H6	109.5	H15A—C15—H15B	107.9
C8—C7—C5	121.3 (6)	N1—C16—C15	110.7 (5)
C8—C7—H7	119.4	N1—C16—H16A	109.5
C5—C7—H7	119.4	C15—C16—H16A	109.5
C7—C8—C14	119.7 (6)	N1—C16—H16B	109.5
C7—C8—H8	120.1	C15—C16—H16B	109.5
C14—C8—H8	120.1	H16A—C16—H16B	108.1
N1—C9—C14	107.8 (5)	N1—C17—H17A	109.5
N1—C9—C10	115.3 (5)	N1—C17—H17B	109.5
C14—C9—C10	112.4 (5)	H17A—C17—H17B	109.5
N1—C9—H9	107.0	N1—C17—H17C	109.5
C14—C9—H9	107.0	H17A—C17—H17C	109.5
C10—C9—H9	107.0	H17B—C17—H17C	109.5

### Hydrogen-bond geometry (Å, º)

**Table:**

*D*—H···*A*	*D*—H	H···*A*	*D*···*A*	*D*—H···*A*
O1—H1···O3^i^	0.84	1.96	2.757 (6)	159
O3—H3···O2	0.84	2.17	2.629 (6)	114

Symmetry code: (i) −*x*, *y*+1/2, −*z*+3/2.
